# Prehospital emergency response and management of pregnancy-associated haemorrhage in KwaZulu-Natal Province, South Africa: A Retrospective Cross-Sectional Study

**DOI:** 10.1016/j.afjem.2025.100912

**Published:** 2025-11-06

**Authors:** S Govender, OP Khaliq, T Abel, J Moodley

**Affiliations:** a KwaZulu-Natal College of Emergency Care, Durban, South Africa; b Department of Paediatrics and Child Health, School of Clinical Medicine, Faculty of Health Sciences, University of the Free State, Bloemfontein, South Africa; c Department of Otorhinolaryngology, School of Clinical Medicine, College of Health Sciences, University of KwaZulu-Natal, Durban, South Africa; d Women’s Health and HIV Research Group, Department of Obstetrics and Gynaecology, School of Clinical Medicine, College of Health Sciences, University of KwaZulu-Natal, Durban, South Africa

**Keywords:** Emergency medical services, Emergency care providers, Pregnancy-associated haemorrhage, KwaZulu-Natal Province, Maternal mortality, Prehospital care

## Abstract

**Background:**

Pregnancy-associated haemorrhage (PAH) is a leading contributor to maternal mortality in KwaZulu-Natal (KZN) and the fourth most common cause in South Africa. Delays in treating PAH increase maternal mortality; prompt prehospital response is therefore critical to improve outcomes. The aim of this study was to analyse response times and clinical management of PAH by public sector Emergency Care Providers in KZN, looking specifically at postpartum haemorrhage (PPH).

**Methods:**

A retrospective cross-sectional study was conducted in two phases. Phase 1 involved analysis of emergency call centre records (n = 4779) assessing response time patterns. Phase 2 analysed randomly selected PPH cases (n = 61) to assess clinical management practices. Descriptive statistics summarised demographics, response patterns, and clinical practice, Pearson correlation examined the relationships between time variables, and chi-square tests assessed associations between clinical variables (p < 0.05).

**Results:**

More than half (51.5 %) of PAH cases had ambulance response times >60 min. Median pre-response time was 30 min; overall response time was 63.5 min but both were positively skewed by extreme delays, with higher trimmed means(50.9 min and 85.7 min respectively). Most cases (81.6 %, n = 3899) were transported from primary healthcare facilities to hospitals, with Intermediate Life Support Providers managing 75.7 % of the cases. In the sampled PPH patients, compliance with vital signs monitoring was initially high (91.8 %) but declined in transit (42.6 %). Providers were largely non-compliant in documenting blood loss and patient history. Despite almost all patients showing clinical signs of shock 44.3 % of PPH cases received no intravenous fluids.

**Conclusion:**

Significant time delays in EMS response and inconsistencies in PAH clinical management by EMS providers were identified. Improved resource allocation, focused training and adherence to clinical and departmental guidelines are vital to strengthening maternal emergency care in KZN.

## Abstract (author highlights)

• Obstetric haemorrhage, particularly post-partum haemorrhage, remains a leading direct cause of maternal mortality in sub-Saharan Africa where access to emergency care is limited due to lack of transport, poor infrastructure and rurality.
• There is limited research regarding EMS response and clinical management of obstetric emergencies in an African setting despite a lack of transport being a key contributor to maternal mortality.
• Findings of the current study highlight gaps in response times and the immediate clinical management that can be mitigated through training, policies and strategic allocation of resources (ambulances).
• Lessons learnt from this study can also inform efforts to improve maternal outcomes in other resource-limited African EMS systems.

## African relevance

• Pregnancy-associated haemorrhage, particularly postpartum haemorrhage, remains a leading direct cause of maternal mortality in sub-Saharan Africa where access to emergency care is limited due to lack of transport, poor infrastructure and rurality.
• There is limited research regarding EMS response and clinical management of obstetric emergencies in an African setting despite a lack of transport being a key contributor to maternal mortality.
• Findings of the current study highlight gaps in response times and the immediate clinical management that can be mitigated through training, policies and strategic allocation of resources (ambulances).
• Lessons learnt from this study can also inform efforts to improve maternal outcomes in other resource-limited African EMS systems.

## Introduction

Maternal mortality remains a global burden, with approximately 800 deaths per day, most occurring in low- or middle-income countries (LMIC) [1]. While Obstetric Haemorrhage (OH) is the leading direct cause of maternal death globally [1], it is also a significant challenge in South Africa (SA) with the KwaZulu-Natal (KZN) province being the most affected [2]. One of the factors contributing to maternal deaths is delay in women receiving appropriate care which Thaddeus and Maine [3] stated, occurred in 3 phases: (i) delay in the decision to seek care; (ii) delay in reaching a healthcare facility; and (iii) delay in getting adequate care at the healthcare facility [4].

Delays in reaching facilities are often due to pregnant women walking long distances from rural homesteads and limited transport options [5], with similar findings reported in Ghana [6] and Mozambique [7]. In SA, the National Committee on Confidential Enquiries into Maternal Deaths (NCCEMD) [2] documented delays across all three phases, highlighting lack of emergency transport (phase 2) as a key avoidable factor. These delays are compounded by poor rural road networks [8] and inequalities in health infrastructure and public services in SA [9].

Emergency Medical Services (EMS) are integral in responding to, treating and transporting pregnant women with haemorrhage in pregnancy to appropriate healthcare facilities. This role is aligned to the World Health Organization’s (WHO) drive to improve access to “the most disadvantaged, marginalised and hard-to-reach populations, to ensure that no one is left behind” [10]. However, Govender et al. [11] reported a constrained EMS system in SA, with suboptimal components delaying access to care, consistent with other KZN studies linking ambulance shortages to poor response times [12,13].

In response, the SA Department of Health introduced strategies such as the Campaign on Accelerated Reduction of Maternal (and Child) Mortality in Africa (CARMMA), advocating dedicated obstetric ambulances. However, implementation in KZN has been inconsistent due to staffing and vehicle shortages [11].

Given these challenges and limited research specific to this field, this study was designed to focus on OH, a late pregnancy complication. Preliminary data, however, revealed high numbers of early pregnancy complications prompting a broader investigation into pregnancy-associated haemorrhage (PAH), which encompasses haemorrhage in all the trimesters of pregnancy and the postpartum period. Accordingly, the aim of this research was to analyse prehospital response times and clinical management of PAH, by EC providers in KZN Province of SA. While the research broadly considered PAH, the assessment of clinical management focused on postpartum haemorrhage (PPH) as a clinically significant and commonly encountered subset of PAH for detailed analysis.

## Methodology

### Study design and setting

This was a cross-sectional study using records review to analyse cases of PAH managed by public sector EC providers in KZN between January 2019 to June 2021. The study period spans pre-, intra- and post-COVID-19 phases. PAH was recognised as bleeding in all three trimesters of pregnancy [2].

KZN, one of the provinces in SA, spans 94,361 km² and has the second-largest population (12 312 712) [14]. The province comprises 11 districts, each containing a mix of rural and urban populations [15]. While all districts include both settlement types, 9 are predominantly rural, whereas eThekwini and uMgungundlovu are predominantly urban [15]. Rural areas are defined as low-density municipalities with limited infrastructure and agricultural economies, while urban areas have higher density, developed infrastructure, and diverse economies. This rural–urban mix is relevant for interpreting EMS response patterns and transport times [15].

Each district has both field EMS stations and an Emergency Call Centre (ECC), all staffed by EC providers working 12-hour shifts (07:00–19:00 and 19:00–07:00) [16]. Each telephone call is time-stamped on a vehicle-control form (VCF) that tracks the patients’ transit to hospital. Completed VCFs are entered into one of three electronic database subsets: (i) general cases, (ii) principally inter-facility transfers, and (iii) obstetric cases attended to by dedicated “obstetric ambulances.”

### Study population

The study population comprised all PAH cases attended by public-sector EMS in KZN during the study period. Prehospital care was delivered by EC providers registered with the Health Professions Council of South Africa (HPCSA) at one of three levels: Basic Life Support (BLS), Intermediate Life Support (ILS), or Advanced Life Support (ALS), as detailed in online supplementary material A. These levels correspond to distinct capabilities relevant to PAH management; for example, BLS providers are not authorised to administer intravenous fluids or medications, ILS and ALS providers may initiate fluid resuscitation, and only ALS providers are permitted to administer uterotonics such as oxytocin.

### Sampling strategy

A multiphase (two-phase) sampling strategy was employed in this study.

Phase 1**:** During the study period, 10017 PAH-related ECC records were identified. **Inclusion criteria** were interfacility-transfer (IFT) cases with complete sequential timestamps (call received and dispatch) and patient details. **Exclusion criteria** were records that did not meet these requirements. Using purposive sampling, 4779 IFT cases were selected, as these were clinician-reported rather than call-centre diagnosed, providing greater diagnostic reliability. This subset was considered most appropriate for analysing response times and diagnostic patterns.

Phase 2**:** These 4779 cases were stratified into eight haemorrhage categories. Using purposive sampling, the PPH stratum (210 cases) was selected, given its status as the leading cause of PAH-related maternal death in KZN. Focusing on PPH allowed for an in-depth assessment of clinical management, as analysing all eight PAH categories would have increased complexity and reduced feasibility due to differing clinical protocols. From this stratum, a proportionate, computer-generated simple random sample of 61 records was drawn across six participating districts, ensuring clinical relevance, geographic representation, and a manageable workload for manual data retrieval and review.

### Data collection

Data were obtained from 6 of the 11 districts in KZN (uThukela, uMzinyathi, King Cetshwayo, uMgungundlovu, Ugu, and iLembe). The remaining 5 districts were excluded due to the absence of electronic case records.

Phase 1: Each district ECC used a standardized electronic database to record cases. Data that required analysis included the pre-response time (PRT): calculated from time the call was received to the dispatch of the ambulance and response time (RT): calculated from the time the call was received to the arrival of the ambulance on-scene [17]. Data were verified before being transferred to SPSS v29 for analysis. Additionally, cases were grouped into <20 weeks (early pregnancy haemorrhage) and >20 weeks (late pregnancy haemorrhage).

Phase 2: District staff scanned and submitted 61 randomly selected PPH patient report forms to the research team. Forms were reviewed for key indicators: maternal history, blood loss estimation, initial and continuous vital signs, fluid resuscitation relative to shock status, provider level, and documentation. The review focused on EMS care from loading at the referring facility to handover at the receiving hospital.

### Data and statistical analysis

Data were analysed in Microsoft Excel and SPSS v29. Descriptive statistics (means, medians, frequencies) summarised demographics, response intervals, and clinical management. Pearson’s correlation tested the relationship between pre-response and total response times, and Chi-square tests examined associations between provider qualification and compliance (p < 0.05).

Shock Index (SI) was classified as <0.6 (no shock), 0.6–0.9 (compensated), 1.0–1.4 (mild), 1.5–1.9 (moderate), and ≥2.0 (severe) to grade PPH severity and assess adequacy of prehospital care. Interventions including vital sign monitoring, IV access, fluids, medication, and documentation were benchmarked against national maternity guidelines, HPCSA EMS protocols, and WHO standards to evaluate alignment with patient condition and EC provider level of care.

### Ethical and health regulatory approvals

Ethics approval for the study was obtained from the University of KwaZulu-Natal Biomedical Research Ethics Committee (BREC) (ref.no. BREC/00,003,780/2022) and the KwaZulu-Natal Provincial Department of Health (ref.no. KZ_202,205_008).

## Results

Between January 2019 and June 2021, there were 10 017 cases of PAH recorded in the study districts. These formed the basis for the analysis. From this dataset, 4 779 interfacility transfer (IFT) cases were identified for detailed review. Phase 1 analysed response trends and causes of PAH while Phase 2 analysed clinical management specific to PPH. Broader three-year data (January 2019–December 2021) is provided in the online supplementary B to offer contextual background without interrupting the flow of the main results.

### Phase 1 – Response trends and PAH causes

Some 4779 cases of PAH were transported to healthcare facilities, during the study period. Among these patients, 54 % (n = 2577) had a gestation of <20 weeks, 17 % (n = 822) >20 weeks, and 29 % (n = 1380) indeterminate. The mean age of patients across all cases was 27 years (range: 13 – 48). Miscarriage accounted for 53% (n = 2535) of the cases and was the leading cause of PAH, followed by APH with 10.3% (n = 492), PPH with 4.4% (n = 210) and indeterminate group with 28.9% (n = 1380) (Table 1).

**Table 1:** Causes of pregnancy-associated haemorrhage attended by Emergency Care providers from January 2019 to June 2021 (n = 4779).

**Cause and gestation**	**2019**	**2020**	**2021** **(until June)**	**Total**
**< 20 weeks**				
Ruptured ectopic pregnancy	17 (0.4)	18 (0.4)	7 (0.1)	42 (0.9)
Miscarriage	1002 (21)	1050 (22)	483 (10)	2535 (53)
**> 20 weeks**				
Antepartum haemorrhage	180 (3.8)	219 (4.6)	93 (1.9)	492 (10.3)
Ruptured uterus	2 (0.04)	4 (0.08)	2 (0.04)	8 (0.2)
Vaginal birth trauma (3rd/4th degree tear)	16 (0.3)	11 (0.2)	8 (0.2)	35 (0.7)
Postpartum haemorrhage	82 (1.7)	86 (1.8)	42 (0.9)	210 (4.4)
Retained placenta	25 (0.5)	38 (0.8)	14 (0.3)	77 (1.6)
**Unknown**				
Indeterminate bleeding	502 (10.5)	622 (13)	256 (5.4)	1380 (28.9)
**Total**	1826 (38.2)	2048 (42.9)	905 (18.9)	4779 (100)

Of the cases, 18.4 % (n = 880) were interhospital transfers, while 81.6 % (n = 3899) were from primary health facilities (clinics or community health centres) to hospitals. Most patients (75.7 %, n = 3618) were managed by ILS providers, with 20.7 % (n = 990) by BLS and 3.6 % (n = 173) by ALS.

**Fig. 1:**
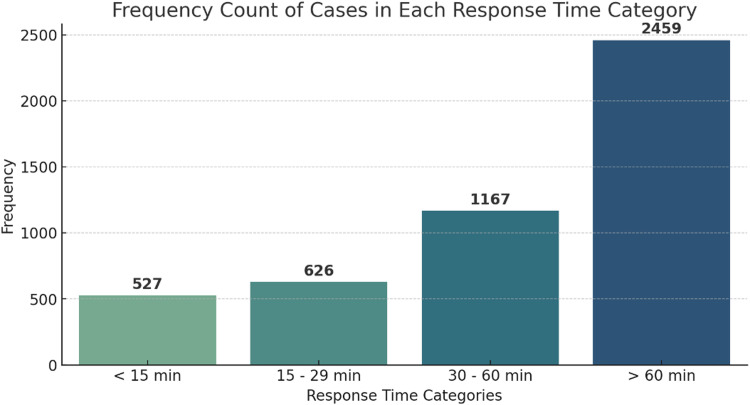
Response time categories for pregnancy-associated haemorrhage.

Fig. 1 shows the distribution of ambulance response times (n = 4779). In 51.5 % (n = 2459) of the PAH cases, ambulances took >60 min (response time) to reach the scene. Only 11 % (n = 527) of the ambulances reached the scene within 15 min.

The pre-response time (PRT) had a median of 30 min (range: 1–609) with a 5 % trimmed mean of 50.9 min, indicating a positively skewed distribution influenced by extreme outliers. The overall response time (RT) showed a similar pattern, with a median of 63.5 min (range: 3–683) and a 5 % trimmed mean of 85.7 min. These wide ranges and skewness likely reflect operational challenges, including ambulance shortages, long interfacility transfer distances, and occasional data logging errors, which inflate the upper extremes. Pearson correlation analysis revealed a strong association between PRT and RT (r = 0.929, p < 0.01).

Across the 6 districts (Table 2), King Cetshwayo recorded the longest median PRT of 39 min (IQR 87) and a median RT of 79 min (IQR 102). In contrast, uThukela had the shortest median pre-response time of 24 min (IQR 58) and a median response time of 53 min (IQR 75) .

**Table 2:** Response trends for cases of pregnancy-associated haemorrhage attended by Emergency Care providers from January 2019 to June 2021 (n = 4779).

**District**	**Total cases (n)**	**Times**	**Mean**	**Median**	**Percentile 25**	**Percentile 75**	**IQR**
iLembe	721	Pre-Response time	53.42	30.00	9.00	78.00	69.00
		Response time	72.95	52.00	24.00	102.00	78.00
King Cetshwayo	1561	Pre-Response time	71.85	39.00	13.00	100.00	87.00
		Response time	103.78	79.00	40.00	142.00	102.00
Ugu	715	Pre-Response time	61.19	33.00	10.00	89.00	79.00
		Response time	87.26	64.00	37.00	117.00	80.00
uMgungundlovu	302	Pre-Response time	68.60	30.00	10.00	92.00	82.00
		Response time	98.19	67.00	30.00	136.00	106.00
uMzinyathi	301	Pre-Response time	49.81	26.00	7.00	64.00	57.00
		Response time	79.17	63.00	30.00	100.00	70.00
uThukela	1179	Pre-Response time	49.78	24.00	9.00	67.00	58.00
		Response time	72.61	53.00	23.00	98.00	75.00

### Phase 2 – Clinical management of PPH

Some 61 cases of PPH were retrieved for analysis, with mean age 25 years; 73.8 % (n = 45) were transferred by EC providers from primary health care facilities to hospital and 26.2 % (n = 16) were transferred between hospitals.

In Phase 2, each patient report form was manually reviewed to assess prehospital actions: vital signs, IV access, fluid resuscitation, medication administration, and documentation of blood loss against the relevant standards outlined in the National Maternity Guidelines , EC provider Clinical Practice Guidelines and WHO recommendations. Patient assessment was evaluated across four indicators: (i) maternal history, (ii) estimation of blood loss, (iii) initial vital signs, and (iv) continuous vital signs monitoring. Treatment was assessed by fluid administration relative to shock status, determined by the Shock Index.

Among PPH patients, 88.5 % (n = 54) of EC providers were non-compliant in obtaining patient history and 96.7 % (n = 59) in estimating blood loss (Table 3). While 91.8 % (*n* = 56) assessed initial vital signs, ongoing monitoring in transit was absent in 57.4 % (n = 35). Blood loss was described as bleeding (77 %; n = 47), severe bleeding (11.5 %; n = 7), bleeding with clots (9.8 %; n = 6), and profuse bleeding (1.6 %; n = 1).

**Table 3:** Patient assessment for Post Partum Haemorrhage (n = 61).

**Clinical assessment indicator**	**Compliance**	**n( %)**	**p-value**
Maternal history	Compliant	7 (11.5)	< 0.001
	Non-Compliant	54 (88.5)	
Estimated fluid loss	Compliant	2 (3.3)	< 0.001
	Non-Compliant	59 (96.7)	
Vital signs	Compliant	56 (91.8)	< 0.001
	Non-Compliant	5 (8.2)	
Continuous vital signs monitoring	Compliant	26 (42.6)	0.249
	Non-Compliant	35 (57.4)	

Almost all - 98.4 % (n = 61) of the PPH patients were in shock and treated by BLS providers (19.7 %, n = 12), ILS providers (70.5 %; n = 43) and ALS providers (9.8 %; n = 6) (Table 4). Most of the patients (68 %; n = 42) were in compensated shock with a shock index of 0.6 – 0.9. Fluid administration occurred in 55.7 % of the patients transported to health care facilities leaving 44.3 % that were transported without intravenous fluid.

**Table 4:** Fluid administration based on shock severity for Post Partum Haemorrhage (n = 61).

		**Shock severity**					
		No Shock	Compensated Shock	Mild Shock	Moderate Shock	Severe Shock	Total
Fluid admin.	Compliant	1 (1.6)	24 (39.3)	8 (13.1)	1 (1.6)	0 (0)	34 (55.7)
	Non-Compliant	0 (0)	18 (29.5)	6 (9.8)	3 (4.9)	0 (0)	27 (44.3)
Total		1 (1.6)	42 (68.9)	14 (24)	4 (6.6)	0 (0)	61 (100)

No shock: <0.6; compensated shock: 0.6 to 0.9; mild shock:1.0 to <1.4; moderate shock: 1.5 to 1.9; severe shock: 2.0.

## Discussion

The study set out to analyse records regarding the response and management of PAH in KZN. Response times data was first analysed (phase 1). The mean patient age was 27 years, outside classical high-risk periods of adolescence and advanced maternal age (≥35 years) [18,19]. Despite this low-risk profile, 54 % presented with early pregnancy bleeding (<20 weeks), associated with complications such as premature birth, APH, and miscarriage [20]. Sayyad et al. [20] found that over 50 % of early pregnancy bleeding cases resulted in pregnancy loss, consistent with our finding that miscarriage accounted for 53 % of cases.

Among PAH >20 weeks, APH (10.3 %; n = 492) and PPH (4.4 %; n = 210) appeared marginal, but many cases were likely included in the “indeterminate” group (28.9 %; n = 1380), recorded only as “per vaginal bleeding.” Proper diagnosis would reclassify and increase the cases into the <20 week or >20 week groups. Diagnostic gaps across districts highlight inadequate ECC history-taking and warrant further investigation.

Most PAH cases (81.6 %, n = 3899) were transferred from clinics and CHCs to hospitals, reflecting adherence to SA’s referral system [21]. Similar patterns were reported in India [22]. Despite SA’s robust referral architecture [23], EMS coverage across sub-Saharan Africa remains limited, with fewer than one-third of countries reporting formal prehospital services [24,25].

District EMS response varied, likely due to ambulance availability, workload, and geography. King Cetshwayo had the longest PRT (39 min) and RT (79 min), with some cases exceeding 120 min, whereas uThukela had the shortest PRT (24 min) and RT (53 min), reflecting more efficient deployment. Ambulance shortages as a key contributor to prolonged PRT are consistent with other KZN studies [[11], [30]], though other contributing factors require further exploration [[12], [26]]. With 51.5 % (n = 2459) of cases exceeding 60 min (provinical norm) [27], EMS response is delayed, supporting NCCEMD reports [2,28]. Similar delays were observed in Gauteng [28], while Sierra Leone reported shorter PRTs (14 min) but similar RTs due to road and distance constraints [29].

Phase 2 findings were interpreted within the EMS phase of care, from patient loading at the referring facility to handover at the receiving facility. The first set of vital signs provided a baseline to evaluate whether interventions, such as fluid administration or ongoing monitoring, were appropriately initiated or continued during transport. Pre-transfer care was not assessed; the focus remained on identifying lapses within EMS responsibility during transfer.

This study focused on interfacility transfers (IFTs), reflecting care in a referral-based context. IFT records were selected for their completeness and confirmed diagnoses, though prior treatment cannot be assumed adequate. Many patients remained in shock during transfer, and essential interventions, such as fluid administration, were often missed, highlighting gaps in prehospital care. The cohort’s mean age was 25 years. Phase 1 described system-level response trends across all PAH types, while Phase 2 purposively focused on PPH, a leading cause of maternal death. This purposive narrowing may introduce sampling bias and limits generalisability but enabled focused clinical analysis. Prehospital care quality was assessed using five indicators benchmarked against national maternity and EMS guidelines: maternal history, blood loss estimation, initial and continuous vital signs, and intravenous fluid administration aligned to shock severity.

Most EC providers recorded an initial set of vital signs, but no further clinical assessments were documented during transport. Providers were largely non-compliant in recording maternal history, estimating blood loss, and monitoring vital signs continuously. Similar gaps were reported in another KZN IFT study, where no action was taken when patients’ conditions changed [30]. Because PPH signs such as blood loss, tachycardia, and hypotension may be masked, continuous in-transit assessment is essential for timely symptomatic treatment [31]. Delayed initiation of guideline-based PPH interventions in the prehospital setting remains a recognised contributor to preventable maternal mortality [32].

Most PPH patients (98.4 %, *n* = 61) transferred to higher-level facilities were in shock (Table 4), yet referring clinics did not initiate intravenous fluids in 27 (44.3 %) cases. Of these, 15 were managed by ILS and ALS providers who also failed to administer fluids despite it being within their level of care, while the remaining 12 (19.7 %) were transferred by BLS providers, who are not authorised to establish intravenous access under HPCSA regulations. Similarly, Moores [33] reported that EC providers in Timor Leste did not provide intravenous fluids during PPH IFTs. These omissions contravene national prehospital maternity care guidelines and HPCSA clinical standards [21,34].

In summary, this study found that prehospital PAH care was marked by delayed response times, with many cases exceeding recommended targets, and significant gaps in patient assessment, monitoring, and treatment. Inadequate ECC history-taking contributed to the “indeterminate” group, reflecting non-compliance or limited clinical knowledge, while documentation gaps and inconsistent adherence to EMS responsibilities highlighted systemic and clinical weaknesses.

Addressing these deficiencies requires both operational and clinical reforms. Expanding ambulance availability can reduce prolonged PRTs, and strengthening provider capacity through obstetric-focused training, guideline reinforcement, and scenario-based refreshers will improve assessment and interventions. Routine audits and supervision are also recommended to ensure consistent documentation and adherence to standards.

### Strengths and limitations

This study used the largest known EMS dataset on PAH, spanning rural and urban districts, with standardised VCFs providing detailed, time-stamped operational data rarely available in obstetric EMS research. However, findings are limited to the public-sector EMS in KZN, with five districts excluded due to poor-quality records and reliance on retrospective documentation. The Phase 2 PPH sample was small and not representative of other haemorrhage types, and analyses were restricted to interfacility transfers. The absence of pre-transfer timelines and treatment histories prevented determination of the exact stage of care, so results reflect only care documented during the EMS phase.

## Conclusion

This study highlights system-level inefficiencies and clinical shortcomings limiting EMS response to PAH in KZN. By targeting ambulance availability, provider skills, monitoring, and intervention practices, policymakers and EMS managers can strengthen prehospital care and align with national health guidelines. Such reforms are urgently needed to improve timeliness and quality of obstetric emergency care and reduce maternal morbidity and mortality in the province.

## Author contributions: CReDiT statement

**S Govender:** Conceptualization, Methodology, Investigation, Resources, Writing – original draft. **OP Khaliq:** Project administration, Visualization, Writing - review & editing. **T Abel:** Data curation, Validation, Writing- review & editing. **J Moodley:** Supervision, Validation, Writing- review & editing.

## Dissemination of results

The result of this study will be presented to the National School of Government online research presentations, KZN Provincial Emergency Medical Service Committee and the KZN College of Emergency Medical Care.

## Declaration of competing interest

There is no conflict of interest to declare.

## Appendix Supplementary materials

Image, application 1

Image, application 1

## Footnotes

Supplementary material associated with this article can be found, in the online version, at 10.1016/j.afjem.2025.100912.

## References

[1] World Health Organization (2023). https://www.who.int/publications/i/item/9789240085398.

[2] National Committee for Confidential Enquiry into Maternal Deaths (2024).

[3] Thaddeus S., Maine D. (1994). Too far to walk: maternal mortality in context. Soc Sci Med.

[4] Alam N., Chowdhury M.E., Kouanda S. (2016). The role of transportation to access maternal care services for women in rural Bangladesh and Burkina Faso: a mixed methods study. Int J Gynaecol Obstet.

[5] Makacha L., Mlambo R., Chikoko L., Martinez-Alvarez M., Makanga P.T. (2022). Concerning mobilising transport for accessing maternal health care and how impactful strategies are in low-resourced settings: a scoping review. The Dyke.

[6] Atuoye K.N., Dixon J., Rishworth A. (2015). Can she make it? Transportation barriers to accessing maternal and child health care services in rural Ghana. BMC Health Serv Res.

[7] Munguambe K., Boene H., Vidler M. (2016). Barriers and facilitators to health care seeking behaviours in pregnancy in rural communities of southern Mozambique. Reprod Health.

[8] Snyman L., Coetzee S. (2024). Measuring geographic accessibility in data poor rural areas by augmenting the road network with a triangular irregular network – A case study in the O.R. Tambo District Municipality of the Eastern Cape, South Africa. J Transp Geogr.

[9] Achoki T., Sartorius B., Watkins D. (2022). Health trends, inequalities and opportunities in South Africa’s provinces, 1990–2019: findings from the Global Burden of Disease 2019 study. J Epidemiol Community Health.

[10] World Health Organization (2015). https://platform.who.int/docs/default-source/mca-documents/qoc/quality-of-care/strategies-toward-ending-preventable-maternal-mortality-(epmm).pdf?sfvrsn=a31dedb6_4.

[11] Govender S., Khaliq O.P., Naidoo R., Moodley J. (2024). The current state of emergency medical services in South Africa: a review. S Afr J Sci.

[12] Govender S. (2011).

[13] Finlayson M.J. (2023).

[14] Statistics South Africa (2024). https://www.statssa.gov.za/publications/P0302/P03022024.pdf.

[15] KwaZulu-Natal Department of Economic Development, Tourism and Environmental Affairs (EDTEA) (2020). https://www.kznedtea.gov.za/documents/KZN%20Rural%20and%20Township%20Economies%20Revitalisation%20Strategy%20-%20Situation%20Analysis%20Report%20-%20December%202020%20(Draft).pdf.

[16] KwaZulu-Natal Department of Health (2024). https://www.kznhealth.gov.za/EMS.htm.

[17] South African Department of Health (2021). https://www.gov.za/sites/default/files/gcis_document/202102/44161gon94.pdf.

[18] Souza J.P., Tunçalp Ö., Vogel J.P. (2024). A global analysis of the determinants of maternal health and transitions in maternal mortality. Lancet Glob Health.

[19] Nyongesa P., Ekhaguere O.A., Marete I. (2023). Maternal age extremes and adverse pregnancy outcomes in low-resourced settings. Front Glob Womens’ Health.

[20] Sayyad H., Jadoon H., Gul M. (2023). Pregnancy outcome in first trimester bleeding. Pak J Med Health Sci.

[21] South African Department of Health (2020). https://knowledgehub.health.gov.za/elibrary/referral-policy-south-african-health-services-and-referral-implementation-guidelines.

[22] Singh S., Doyle P., Campbell O.M.R. (2017). Interfacility transfer of pregnant women using publicly funded emergency call centre-based ambulance services: a cross-sectional analysis of service logs from five states in India. BMJ Open.

[23] Nassoro M.M., Chiwanga E., Lilungulu A. (2020). Maternal deaths due to obstetric haemorrhage in Dodoma Regional Referral Hospital, Tanzania. Int J Reprod Med.

[24] Mould-Millman N., Dixon J., Sefa N., Yancey A., Hollong B., Hagahmed M. (2017). The state of emergency medical services (EMS) systems in Africa. Prehosp Disaster Med.

[25] Okong D.A., Kajjimu J., Kalanzi J., Namirembe M.S., Agaba P.K., Kintu A. (2023). Description and analysis of the emergency obstetric interfacility ambulance transfers (IFTs) to Kawempe National Referral Hospital in Uganda. Afr J Emerg Med.

[26] Jeeana S., Naidoo T.D. (2023). An appraisal of the referral system and outcome for obstetric patients referred to a tertiary centre. Trop Doct.

[27] Van der Net W., Vincent-Lambert C., Govender K. (2023). Emergency medical service response and mission times in an African metropolitan setting. South African Journal of Pre-hospital Emergency Care.

[28] Maswime T.S., Buchmann E. (2017). Near-miss maternal morbidity from severe haemorrhage at caesarean section: a process and structure audit of system deficiencies in South Africa. S Afr Med J..

[29] Caviglia M., Putoto G., Conti A. (2021). Association between ambulance prehospital time and maternal and perinatal outcomes in Sierra Leone: a countrywide study. BMJ Global Health.

[30] KwaZulu-Natal Department of Health (2024).

[31] Wormer K.C., Jamil R.T., Bryant S.B. (2024). StatPearls.

[32] Akter S., Forbes G., Miller S. (2022). Detection and management of postpartum haemorrhage: qualitative evidence on healthcare providers' knowledge and practices in Kenya, Nigeria, and South Africa. Front Glob Womens Health.

[33] Moores J., De Jesus G.A. (2018). Management of post-partum haemorrhage in the Timor Leste National Ambulance Service. Emerg Med Australas.

[34] Fawcus S. (2018). Alerts for managing postpartum haemorrhage. S Afr Med J..

