# Supplementary material for: Prehospital emergency response and management of pregnancy-associated haemorrhage in KwaZulu-Natal Province, South Africa: A Retrospective Cross-Sectional Study
Source: Afr J Emerg Med. 2025 Nov 6;15(4):100912. doi: 10.1016/j.afjem.2025.100912 (PMC12861671; doi:10.1016/j.afjem.2025.100912)
Supplement: Supplementary file 1 [file mmc1.docx]

**Appendix A**

Study Population and Sampling Strategy

The target population comprised all Emergency Care (EC) providers, working on ambulances, employed in the public EMS sector across KwaZulu-Natal (KZN) and registered with the Health Professions Council of South Africa (HPCSA). In South Africa, six categories of registration exist, each associated with varying training durations, academic levels, and clinical scopes of practice. These include: Basic Ambulance Assistant (BAA), Ambulance Emergency Assistant (AEA), Critical Care Assistant (CCA), Emergency Care Assistant (ECA), Emergency Care Technician (ECT), and Emergency Care Practitioner (ECP).

For the purpose of this study, participants were grouped into three main categories reflecting general levels of care and clinical scope:

- Basic Life Support (BLS) – comprising BAA and ECA qualifications, were grouped together as both operate under supervised practice and are not permitted to manage patients independently without oversight from a higher-qualified provider.
- Intermediate Life Support (ILS) – comprising AEA (independent practice)
- Advanced Life Support (ALS) – comprising CCA, ECT, and EC qualifications (independent practice)

Emergency Care Technicians (ECTs) and Emergency Care Practitioners (EC PROVIDERSs) were grouped under ALS due to their expanded scopes of practice, alignment with advanced prehospital care as expressed in the “Regulations Relating Standards for Emergency Medical Services”:

"Advanced Life Support (ALS)" means a level of care provided within the Paramedic, Emergency Care Technician or Emergency Care Practitioner scope of practice as determined by the Health Professions Council of South Africa in terms of the Health Professions Act, 1974 (Act No. 56 of 1974)

We acknowledge that it simplifies a complex qualification structure and may limit nuanced interpretation of some findings. This limitation is noted in the discussion.
