# Supplementary material for: Prehospital emergency response and management of pregnancy-associated haemorrhage in KwaZulu-Natal Province, South Africa: A Retrospective Cross-Sectional Study
Source: Afr J Emerg Med. 2025 Nov 6;15(4):100912. doi: 10.1016/j.afjem.2025.100912 (PMC12861671; doi:10.1016/j.afjem.2025.100912)
Supplement: Supplementary file 2 [file mmc2.docx]

**Appendix B**

**Context**

*Emergency call requests for all cases*

The ECC received 817 284 cases between January 2019 and December 2021, of which 22.9% (n=187 360) were pregnancy related and 77% (629 924) making up the rest of the cases (Table 1).

| **Table B1. Emergency transport requests in KwaZulu-Natal Province between January 2019 and December 2021 (n=817284)** | | | | |
| --- | --- | --- | --- | --- |
| **Variable** | **2019**  **n(%)** | **2020**  **n(%)** | **2021**  **n(%)** | **Total**  **n(%)** |
| General cases | 230573 (28.2) | 209548 (25.6) | 189803  (23.2) | 629924  (77.1) |
|  |  |  |  |  |
| Pregnancy related cases | 64329  (7.9) | 65397  (8) | 57634  (7.1) | 187360  (22.9) |
|  |  |  |  |  |
| Total | 294902  (36.1) | 274945  (33.6) | 247437  (30.3) | 817284  (100) |
|  |  |  |  |  |

*Pregnancy related cases*

Of the 187 360 cases pregnancy related cases, 33.35% (n=62 485) were in labour, 6.4% (n=12065) were PAH, 23.1% (n=43 306) indeterminate, 15.9% (n=29 829) exempt (cancelled) and Reasons for cases being exempt included patients being taken by private transport; patients delivered on-scene; patients not found on-scene.

**Table B2. Pregnancy related cases in KwaZulu-Natal Province between January 2019 and December 2021 (n=187 360)**

| **Pregnancy related cases** | **Total cases**  **2019 - 2021 (n)** |
| --- | --- |
| In labour | 62485 (33.4) |
| Pregnancy-associated haemorrhage | 12065 (6.4) |
| Indeterminate | 43306 (23.1) |
| Exempt (case cancelled) | 29829 (15.9) |
| Other | 39675 (21.2) |
| Total | 187360 (100) |

*Pregnancy-associated haemorrhage and sampling*

Between January 2019 and December 2021, 12 065 cases of pregnancy-associated haemorrhage (PAH) were recorded, providing overall context for the three-year burden. However, as the study’s analysis period was January 2019 to June 2021, only 10 017 cases fell within this timeframe. From these, a sample of 4 779 interfacility transfer (IFT) cases was identified for detailed analysis.
